# Low lymphozyte pool, colon perforation and hydrocephalus as clinical features in an infant with a postzygotic *PIK3CA* variant

**DOI:** 10.3389/fped.2025.1650077

**Published:** 2025-11-20

**Authors:** Ximena Léon-Lara, Sarina Ravens, Sandra von Hardenberg, Bernd Auber, Anke K. Bergmann, Laura Ospina, Florian Guthmann, Christiane Wübbena, Jan Brechler, Jürgen Weidemann, Manuela F. Richter

**Affiliations:** 1 Institute of Immunology, Hannover Medical School, Hannover, Germany; 2 Cluster of Excellence RESIST (EXC 2155), Hannover Medical School, Hannover, Germany; 3 Department of Human Genetics, Hannover Medical School, Hannover, Germany; 4 Clinical Genetics and Genomic Medicine, University Hospital Würzburg, Würzburg, Germany; 5 Department of Neonatology, AUF DER BULT- Children’s and Youth Hospital, Hannover, Germany; 6 Department of Pediatric Neurology, AUF DER BULT- Children’s and Youth Hospital, Hannover, Germany; 7 Department of Pediatric Surgery, AUF DER BULT- Children’s and Youth Hospital, Hannover, Germany; 8 Department of Pediatric Radiology, AUF DER BULT- Children’s and Youth Hospital, Hannover, Germany

**Keywords:** *PIK3CA* gene mutation, severe lymphopenia, intern hydrocephalus, intestinal vascular malformation, necrotizing enterocolitis (NEC)

## Abstract

Pathogenic variants in the *PIK3CA* gene, which encodes the p110-α catalytic subunit of the phosphoinositide 3-kinase (PI3K), are commonly associated with overgrowth syndromes and cancer. We report a patient with the point variant c.1030G>A p.(Val344Met) in the *PIK3CA* gene who presented shortly after birth with viral sepsis and and severe lymphopenia, followed by colonic perforations. Histopathology showed ulcerative necrotizing colitis with lymphatic vascular malformation. The patient subsequently developed hydrocephalus requiring a ventriculoperitoneal shunt, complicated by refractory ascites that resolved with acetazolamide therapy. Awareness of the potential disease spectrum through early molecular diagnosis, combined with a comprehensive immunologic evaluation, enabled individualized management via closer clinical monitoring and timely interventions to prevent and control neurological and infectious complications. This case highlights the phenotypic heterogeneity of *PIK3CA* pathogenic variants and the importance of early precision medicine in pediatric care.

## Introduction

The *PIK3CA* gene encodes the p110-α catalytic subunit of the class I phosphoinositide 3-kinase (PIK), a key enzyme involved in numerous cellular processes, including cell growth, proliferation, survival, metabolism, and angiogenesis (1, 2). Class I PI3Ks promote the phosphorylation of phosphatidylinositol-(4,5)-bisphosphate (PIP2) to phosphatidylinositol-(3,4,5)-triphosphate (PIP3), thereby activating the PIK/AKT/mTOR signaling pathway (2, 3). Somatic variants in the *PIK3CA* gene are among the most prevalent genetic alterations in various human cancers, including breast, colorectal, and cervical cancers, playing a critical role in oncogenesis (1, 2). Beyond cancer, *PIK3CA* variants are associated with a range of rare, non-malignant overgrowth disorders collectively known as PIK3CA-related overgrowth spectrum (PROS) (4, 5). These nonhereditary conditions are caused by postzygotic, somatic variants in the *PIK3CA* gene, which result in a mosaic distribution of affected tissues (4, 6). These variants lead to constitutive activation of the PIK/AKT/mTOR signaling pathway, promoting dysregulated cellular growth and proliferation (3).

The somatic nature of these variants contributes to the variability in phenotypic presentation, which also depends on the timing and location of the variant during development (3, 6). The PROS spectrum ranges from isolated, localized, or segmental overgrowth to syndromic disorders, including the megalencephaly-capillary malformation (MCAP) and the megalencephaly-polymicrogyria-polydactyly-hydrocephalus (MPPH) syndromes (4, 7). Immune dysfunction is rarely reported in PROS, most often in association with lymphatic abnormalities (8). Timely diagnosis of PIK3CA-related disorders and associated complications is essential, given the progressive nature of overgrowth and potential complications (5, 6). Here, we present the clinical course of an infant with a *de novo* postzygotic c.1030G>A p.(Val344Met) variant. The infant initially presented with rotavirus-induced neonatal sepsis, complicated with transient immune dysregulation that lasted several weeks. During follow-up, he developed hydrocephalus requiring a ventriculoperitoneal (VP) shunt, which was complicated by refractory ascites, as well as an intraspinal peripheral nerve sheath tumor.

## Results

### Clinical presentation

A male infant was spontaneously delivered at 37 + 3 weeks of gestation in good general condition (Apgar 9/10/10), with a weight of 2,820 g (20th percentile), and a length of 52 cm (73rd percentile). Delivery occurred seven days after rupture of membranes, with clear amniotic fluid. Pregnancy (gravida 3, para 2) was uncomplicated, except for borderline abnormal fetal and umbilical Doppler findings two weeks before delivery. On examination, macrocephaly (head circumference 38 cm, >99th percentile), hypospadias, and right foot hexadactyly were noted. There was no family history of genetic or neurological diseases.

**Figure 1:**
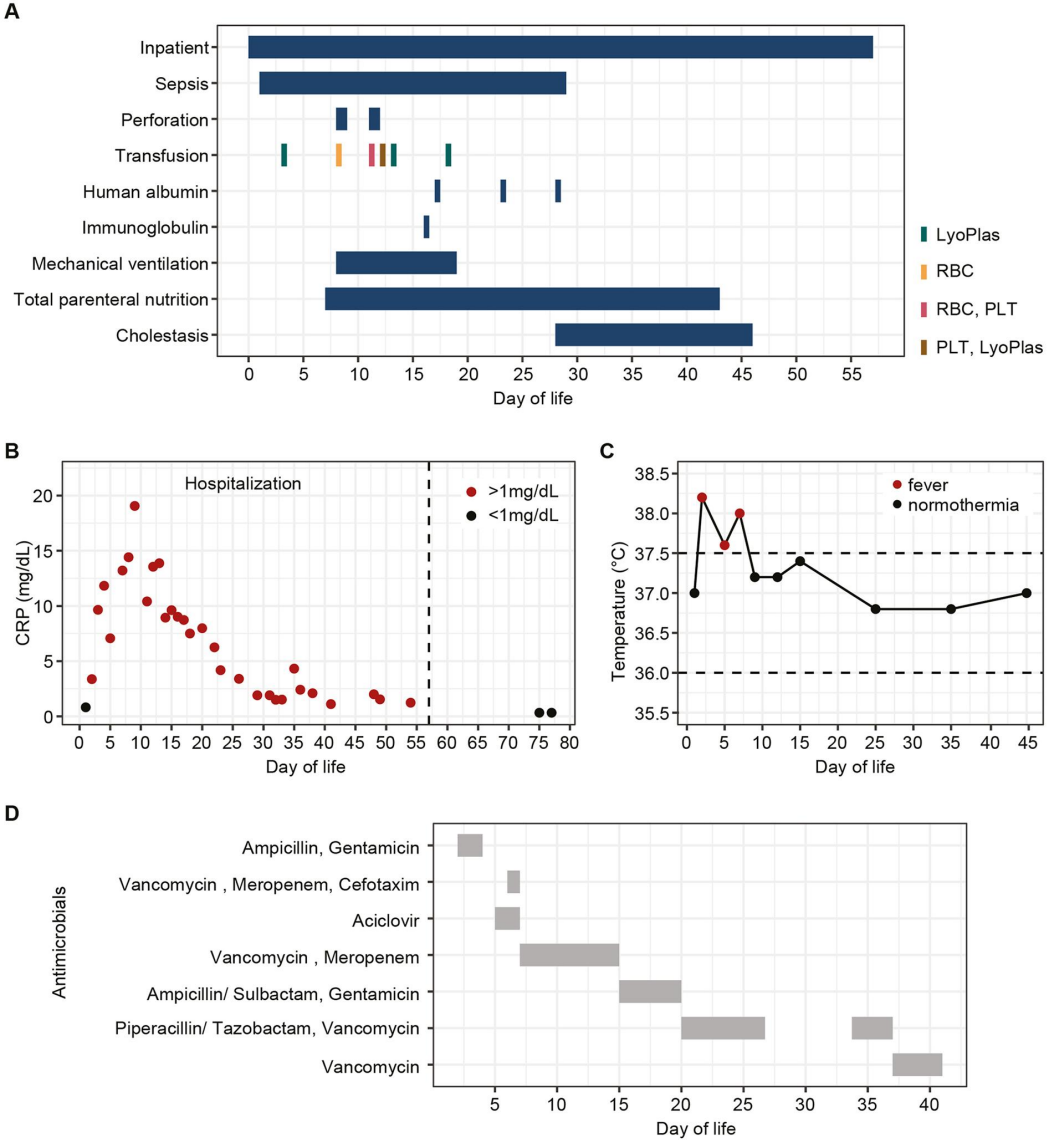
Clinical course and management during initial hospitalization. **(A)** Schematic overview of complications and interventions by day of life. **(B)** C-reactive protein (CRP) levels by day of life, >1 mg/dl (red dots), were considered elevated. **(C)** Body temperatures by day of life; values >37.5°C (red dots) were considered fever. **(D)** Antibiotic and antiviral treatments during hospitalization by day of life. LyoPlas, lyophilized plasma; RBC, red blood cells; PLT, platelets.

**Figure 2:**
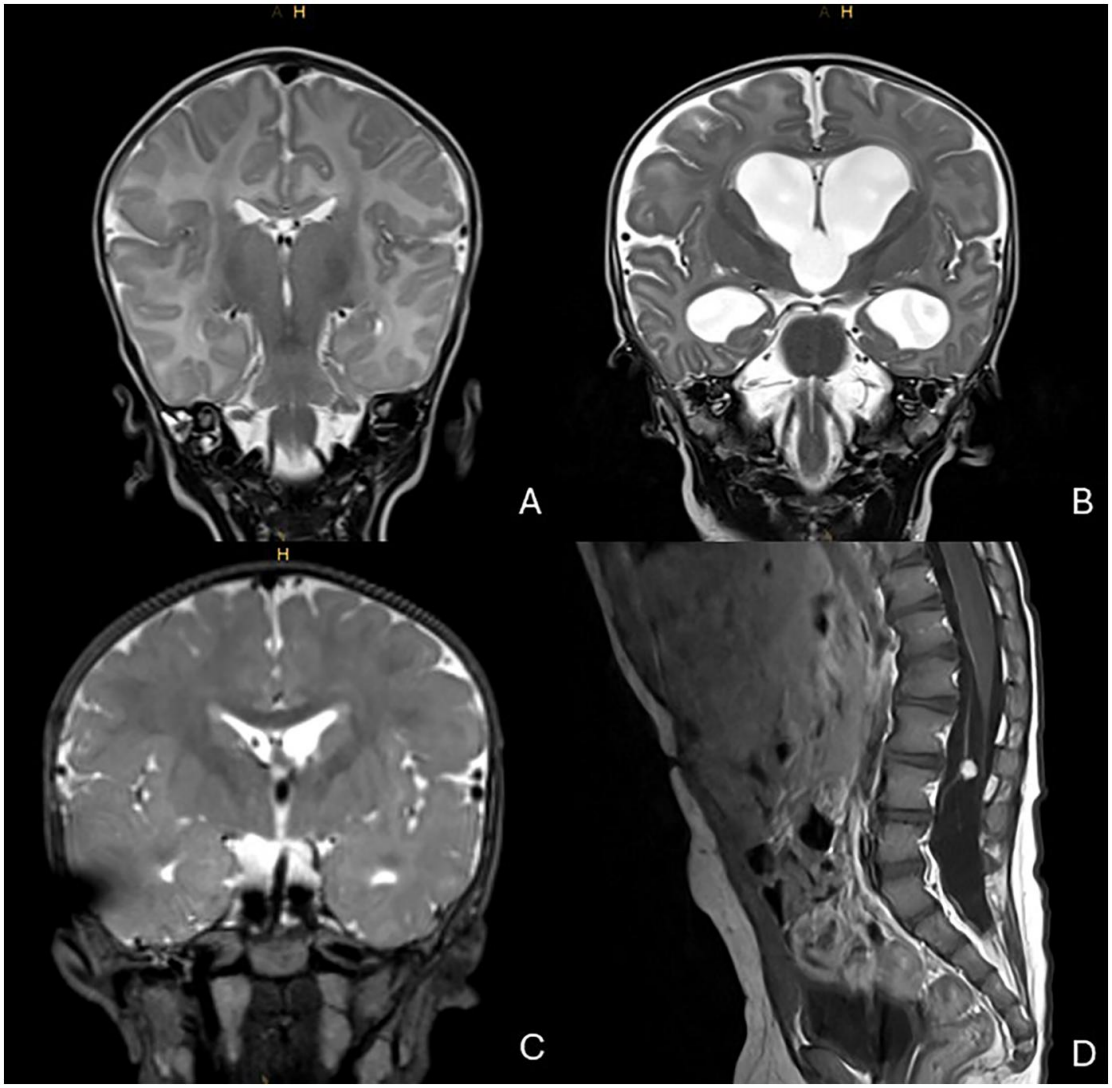
MRI imaging over time. **(A)** T2-weighted MRI during initial hospitalization (1 month, 17 days of age); normal cerebral ventricular width. **(B)** T2-weighted MRI at 6 months, 18 days of age; hydrocephalus with enlargement of ventricles I–III. **(C)** T2-weighted MRI at 1 year, 24 days of age; relief of hydrocephalus via right frontal shunt catheter. **(D)** T1-weighted MRI with contrast at 1 year, 10 months of age; solid nodule with contrast enhancement, stable in size since initial detection at 1 year, 24 days of age.

At 12 h of life, the infant was admitted to the NICU with suspected sepsis. He presented with poor general condition, hypothermia, hypoglycemia, mildly elevated IL-6 [81 pg/ml, (0–50)], and normal initial CRP concentration [0.82 mg/dl, (0–1.0)] (Figures 1A–C). Serial blood cultures at diagnosis were negative, while stool PCR was positive for rotavirus. A sharp rise in the inflammatory markers (IL-6 284 pg/ml and CRP 10.41 mg/dl) prompted broad-spectrum antibiotics (Figure 1D). Respiratory adaptation disorder initially required CPAP, followed by invasive ventilation after colonic perforations on days 8 and 11 (Figure 1A). Surgery revealed an extensive gangrenous perforation involving the transverse and descending colon, requiring partial resection of the transverse colon and creation of an ostomy. Three days later, a second perforation in the transverse colon was treated with a protective terminal ileostomy. Histopathology showed patchy ulcerative necrotizing colitis with a mixed capillary-lymphatic vascular malformation. During hospitalization, the infant required prolonged parenteral nutrition, which caused transient severe cholestasis (Figure 1A). Initial neurological evaluation (EEG, cranial ultrasound, MRI) was unremarkable (Figure 2A).

Ultra-rapid whole genome sequencing (urWGS) identified a *de novo* postzygotic point variant, c.1030G>A, in exon 5 of the *PIK3CA* gene (NM_006218.3). This variant results in a p.(Val344Met) amino acid substitution in the helical domain of the PI3K p110-α subunit and has been previously associated with PROS (6, 9–11). The same variant was also detected by panel sequencing of the resected intestine at a similar allele frequency (48.67% and 47.12%) as in the blood.

### Long-lasting lymphopenia during initial hospitalization

**Figure 3:**
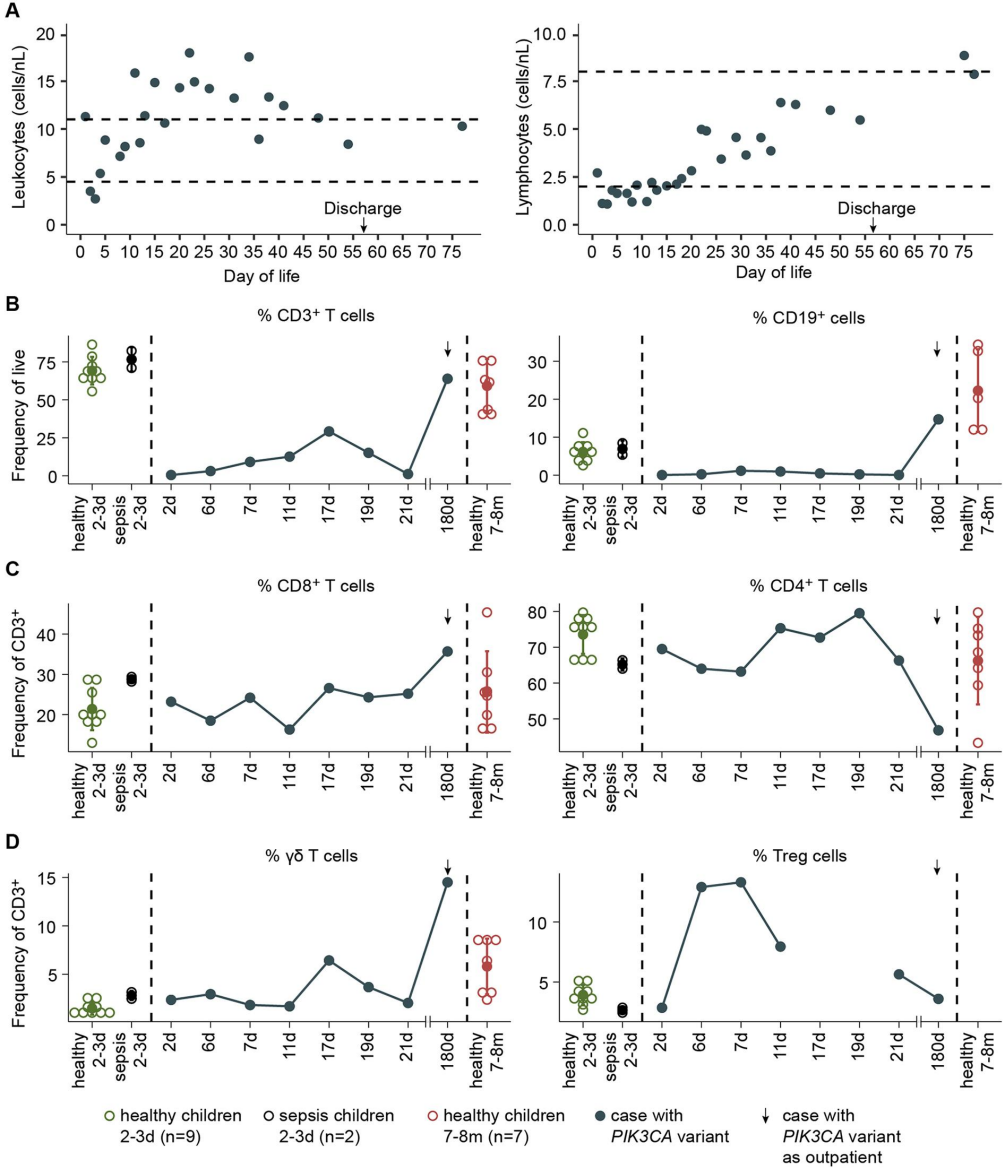
Lymphocyte profile in a patient with *PI3KCA* pathogenic variant. **(A)** Leukocyte and lymphocyte counts by day of life. **(B)** Frequency of CD3T cells and CD19+ cells among live PBMCs from the patient (blue), healthy neonates at 2–3 days (green), neonates with sepsis at 2–3 days (black), and healthy infants at 7–8 months (red). **(C)** Frequency of CD8T cells, CD4T cells, **(D)** γδ T cells, and Treg cells among CD3T cells of the indicated donors.

The infant exhibited persistent lymphopenia (Figure 3A) with markedly reduced B (CD19^+^) and T (CD3^+^) cells compared to neonates with sepsis (Figure 3B). Despite the overall reduction in the T cell pool, the proportions of CD8^+^, CD4^+^, and γδ T cells within the CD3^+^ population were preserved, with a discrete increase in Treg cells (Figures 3C,D). Lymphocyte frequencies normalized after the initial hospitalization (Figure 3B), suggesting a transient alteration of the immune response to acute infection-related stress.

**Figure 4:**
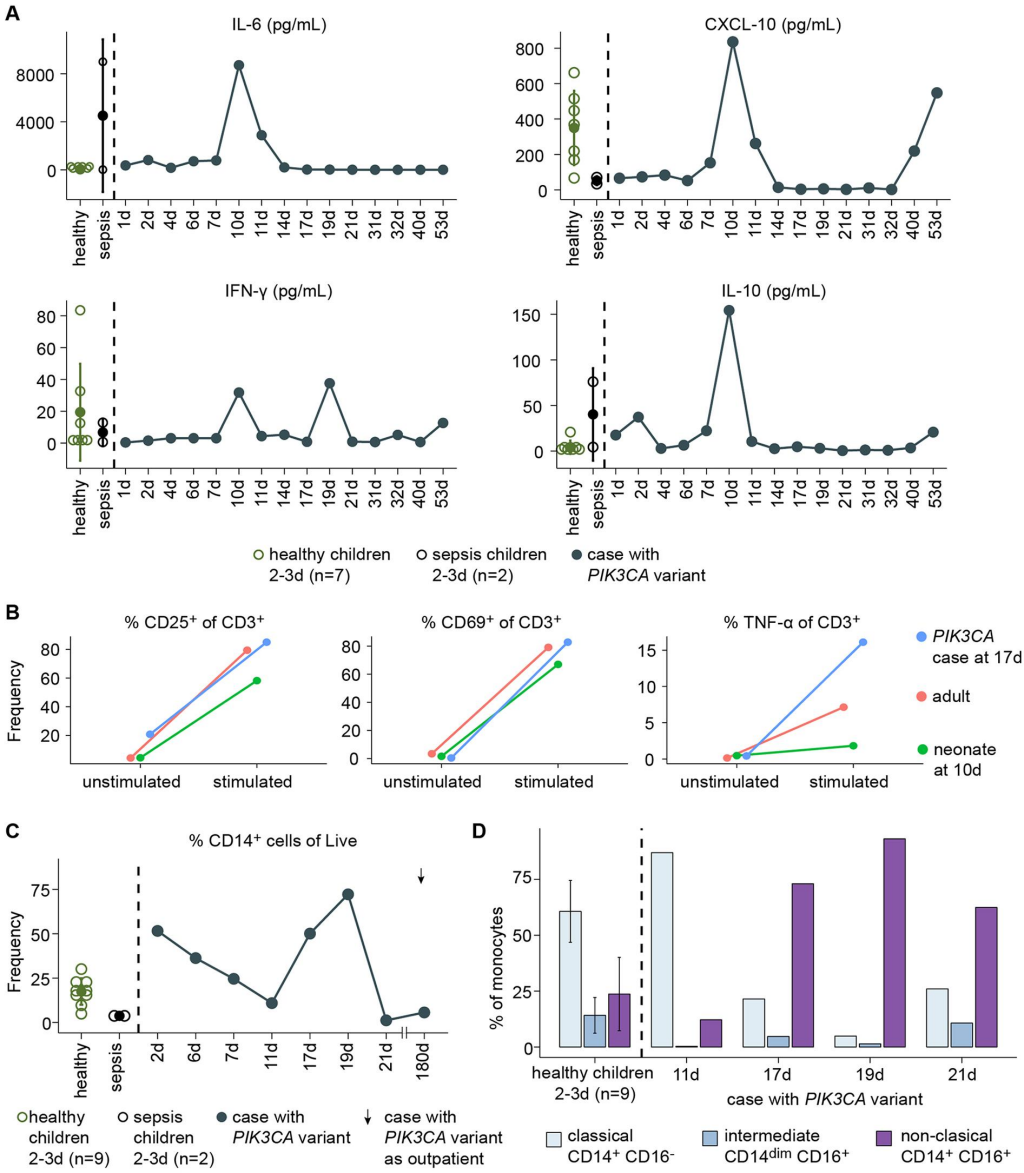
Immune profiling in a patient with *PI3KCA* pathogenic variant. **(A)** Plasma cytokine levels in the patient (blue), healthy neonates at 2–3 days (green), and neonates with sepsis at 2–3 days (black). **(B)** Frequency of CD25, CD69, or TNF-α among CD3T cells following anti-CD3/anti-CD28 stimulation in the patient at 17 days of age (blue), a healthy adult (pink), and a healthy neonate at 10 days of age (green). **(C)** Frequency of monocytes (CD14+) among live PBMCs in the patient (blue), healthy neonates at 2–3 days (green), and neonates with sepsis at 2–3 days (black). **(D)** Immunophenotype of CD14+ cells of the patient and healthy neonates at 2–3 days of age.

On day 10 of hospitalization, during colonic perforation, both pro-inflammatory (IL-6, CXCL-10, IFN-γ) and anti-inflammatory cytokines were elevated (Figure 4A). Meanwhile, CD4^+^ and CD8^+^ T cell functionality remained preserved, with expression of activation markers (CD69 and CD25) and TNF-α production following anti-CD3/anti-CD28 stimulation comparable to controls (Figure 4B). Monocyte frequency (CD14+) increased (Figure 4C) with a shift from classical inflammatory (CD14^+^ CD16^−^) to non-classical anti-inflammatory (CD14^+^ CD16^+^) phenotypes during hospitalization (Figure 4D).

### Follow-up of further complications and neurological involvement

**Figure 5:**
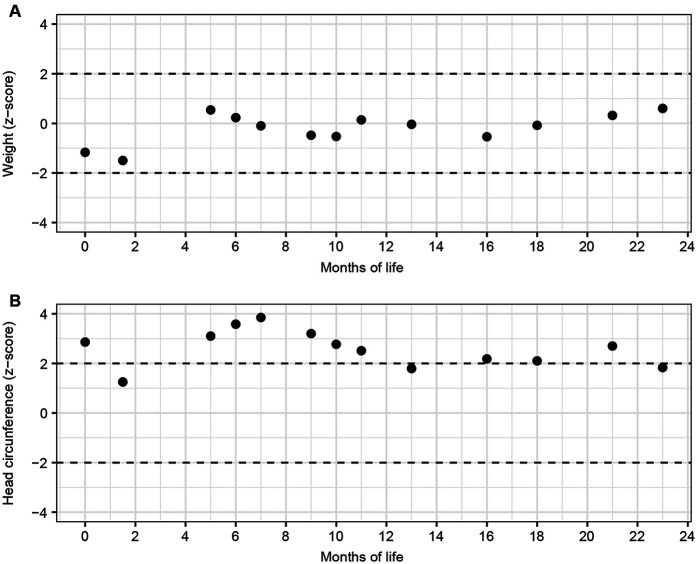
Growth trajectories. *Z*-scores of **(A)** weight and **(B)** head circumference by month. Weight *z*-scores were calculated according to Kromeyer-Hauschild et al., and head circumference according to Braegger et al.

At five months of age, the EEG showed intermittent frontal delta activity and isolated sharp waves, without seizures or infantile spasms. While growth was age-appropriate (Figure 5A), head circumference remained percentile-volatile (Figure 5B). By six months, cranial ultrasound revealed progressive hydrocephalus with enlargement of ventricles I-III, which was confirmed by MRI (Figure 2B). The MRI included a high-resolution, flow-sensitive, thin-slice T2 SPACE sequence, showing an anatomically patent aqueduct and visible CSF flow phenomena. A VP shunt was placed at seven months; however, by 12 months, the patient developed refractory ascites. Serial cerebrospinal fluid (CSF) removal and abdominal punctures were required due to ongoing CSF overproduction (Figure 2C). CSF analysis showed markedly elevated albumin levels [2,520 mg/L, (120–240)], indicating pronounced barrier dysfunction. As an incidental finding during follow-up, a 6 mm diameter benign intraspinal tumor of the nerve roots at the L3/L4 level was detected, with stable size on subsequent evaluations (Figure 2D). From 13 months of age, an off-label therapy trial with acetazolamide significantly reduced CSF and ascites volumes, facilitating motor development. By 15 months, the child achieved independent walking with only mild motor delay. He feeds orally, vocalizes, and performs small tasks. Long-term development outcomes remain to be determined.

## Discussion

Pathogenic *PIK3CA* variants are commonly associated with oncogenesis, overgrowth, and vascular malformations (3, 6). Dysregulated PI3K signaling can also contribute to immune dysfunction, including infection susceptibility and autoimmune manifestations (12). While immunodeficiency is well described in *PIK3CD* variants (12), immune dysregulation has not been previously reported in PROS (6, 11). Here, we present a patient with a pathogenic *PIK3CA* variant who developed prolonged severe neonatal lymphopenia in the context of viral sepsis and peritonitis. The early atypical viral infection, followed by severe peritonitis, may have triggered a transient state of immune dysfunction that resolved once the septic episode subsided. Nevertheless, since PI3K signaling plays a central role in cell proliferation (1, 2, 12), constitutive activation due to the *PIK3CA* variant may have contributed to the lymphopenia and the clinical complications observed. Early detection of the variant through molecular diagnostics, combined with immune monitoring, was crucial for contextualizing, interpreting, and managing these complications. As no further severe infections have occurred, long-term disease progression remains to be determined. Features resembling atypical necrotizing enterocolitis (NEC) were observed in our patient, with histopathology revealing ulcerative necrotizing colitis and a lymphatic vascular malformation. This represents the second reported case of such a presentation in PROS (13), highlighting that, in the neonatal period, PROS can manifest with gastrointestinal disease mimicking atypical NEC, most likely related to the intestinal vascular malformation.

The c.1030G>A p.(Val344Met) variant has been associated with MCAP (9, 10). In this case, macrocephaly was primarily caused by CFS overproduction. Acetazolamide therapy effectively reduced CSF secretion and improved neurologic outcome, highlighting its value when VP shunt treatment is insufficient. Targeted therapies, including PIK inhibitors (e.g., alpelisib) and mTOR inhibitors (e.g., sirolimus), are promising options in PROS by controlling disease progression and improving organ dysfunction (14–16). However, such therapies should be used with caution, particularly in patients with infection susceptibility, given the central role of PI3K in immune signaling (17).

In conclusion, this case expands the phenotypic spectrum of *PIK3CA* pathogenic variants and underscores the importance of multidisciplinary monitoring and careful neurological surveillance. Individualized treatment, including pharmacological CSF reduction and consideration of targeted therapies, may improve outcomes. Further research is needed to clarify the relationship between *PIK3CA* variants and immune dysregulation to guide the safe application of targeted therapies.

## Methods

### Ultra-rapid genome sequencing

EDTA blood was processed using the Illumina DNA PCR-Free Library Preparation Tagmentation Kit, followed by whole genome sequencing (WGS) on an Illumina NovaSeq 6000 sequencer. Sequencing reads were aligned to the human reference genome GRCh38. TruSight™ Software (Illumina, Suite v2.6) and center-specific bioinformatics pipeline were used for alignment, variant calling, variant annotation, filtering, and curation. In addition to variant allele frequency data, prediction tools including phyloP, SIFT, PolyPhen-2, FATHMM, CADD, and REVEL were used. Furthermore, LOVD (https://databases.lovd.nl/shared/), ClinVar (https://www.ncbi.nlm.nih.gov/clinvar), and gnomAD (https://gnomad.broadinstitute.org/) were screened for reported entries of the identified variant. Variant interpretation followed the standards and guidelines of the American College of Medical Genetics and Genomics (18).

### Ethics

Inclusion and sample collection were conducted in accordance with the Declaration of Helsinki and approved by the Institutional Review Board of the Hannover Medical School (No. 10856_BO_K_2023). Written informed consent was obtained from all donors, parents, or guardians in the case of children.

### Peripheral blood mononuclear cell isolation

Peripheral blood mononuclear cells (PBMCs) were isolated by Ficoll-Paque density gradient centrifugation from EDTA blood samples collected at different time points after birth from the studied patient, as well as from nine uninfected healthy neonates (−3 days of age) and two neonates diagnosed with bacterial sepsis (2–3 days of age) at the AUF DER BULT Children's and Youth Hospital, Hannover, Germany. Additionally, PBMCs were isolated from an EDTA blood sample of a healthy adult for *in vitro* assays.

### FACS staining

Freshly isolated PBMCs were incubated for 20 min at room temperature with the following antibodies: anti-CD45 BUV395 (HI30; BD Bioscience), anti-CD3 BUV661 (UCHT1; BD Bioscience), anti-CD8 BUV805 (SK1; BD Bioscience), anti-CD4 BV570 (RPA-T4; BioLegend), anti-CD127 BV650 (A019D5; BioLegend), anti-CD25 PE-Fire700 (M-A250; BioLegend), anti-γδ TCR PE (REA591; Miltenyi Biotec), anti-Vδ2 PerCPVio700 (REA771; Miltenyi Biotec), anti-Vγ9 FITC (REA470; Miltenyi Biotec), anti-Vδ1 VioGreen (REA173; Miltenyi Biotec), anti-CD45RA BV605 (HI100; BioLegend), anti-CD14 BB700 (MφP9; BD Bioscience), anti-CD16 BUV496 (3G8; BD Bioscience), anti-CD56 BUV564 (NCAM16.2; BD Bioscience), anti-CCR7 BV785 (G043H7; BioLegend), anti-CD19 Pe-Cy7 (HIB19; BD Bioscience), anti-IgD BV510 (IA6-2; BD Bioscience), and anti-CD27 Alexa Fluor (O323; BioLegend). Dead cells were detected using Zombi-NIR staining. After washing off excess antibodies, cells were acquired on an Aurora spectral flow cytometer (Cytek) using SpectroFlo v2.2.0 (Cytek). Flow cytometry data were analyzed in Flowjo 10.0 software.

### Cytokine measurement

Plasma cytokines were measured using the LEGENDplex™ Human Essential Immune Response Panel (BioLegend) according to the manufacturer's instructions.

### CD3/Cd28 stimulation

Plates were coated overnight at 4°C with anti-CD3 antibody (BioLegend, #300438) at a final concentration of 4 µg/ml. Freshly isolated PBMCs (0.5  ×  10^6^ cells/ml) were cultured in RPMI-1640 supplemented with 10% heat-inactivated FBS (Sigma), 1% GlutaMAX, 50 µM β-mercaptoethanol, 1% penicillin-streptomycin (all Gibco), and 100 U/ml IL-2. Anti-CD28 antibody was added to the coated wells at a final concentration of 1 µg/ml. Cells were incubated in a humidified CO_2_-incubator at 37°C for 48 h. After incubation cells were stained for 20 min at room temperature with fixable viability dye (Zombie NIR, BioLegend) and the following antibodies: anti-CD45 BUV395 (HI30; BD Bioscience), anti-CD14 BB700 (MφP9; BD Bioscience), anti-CD19 APC-Fire810 (HIB19; Biolegend), anti-CD3 BUV661 (UCHT1; BD Bioscience), anti-TCRγδ PE (REA591; Miltenyi Biotec), anti-CD4 APC (SK3; BD Bioscience), anti-CD8 BV510 (SK1; Biolegend), anti-CD69 BUV737 (FN50; FN50), anti-CD25 PE Fire 700 (M-A251; Biolegend), intracellular anti-TNF-α Alexa Fluor 700 (MAb11; BioLegend), intracellular anti-IFN-γ (B27; BioLegend). Acquisition was performed on an Aurora spectral flow cytometer (Cytek).

## Data Availability

The datasets for this article are not publicly available due to concerns regarding participant/patient anonymity. Requests to access the datasets should be directed to the corresponding author.
